# Mediastinal mature teratoma perforating the right lung that was successfully resected with complete thoracoscopic surgery: A case report

**DOI:** 10.1016/j.ijscr.2018.10.076

**Published:** 2018-11-02

**Authors:** Hiroyasu Matsuoka, Hirochika Matsubara, Aya Sugimura, Tsuyoshi Uchida, Tomofumi Ichihara, Hiroyuki Nakajima

**Affiliations:** Department of Surgery, Faculty of Medicine, University of Yamanashi, 1110 Shimokato, Chuo City, Yamanashi, Japan

**Keywords:** Mediastinal mature teratoma, Thoracoscopic surgery, Asymptomatic, Perforation

## Abstract

**Introduction:**

A perforating mediastinal mature teratoma can cause severe inflammation and adhesion; therefore, open surgery is usually performed. Asymptomatic perforation involving a teratoma is very rare. Complete thoracoscopic surgery is often difficult because of inflammation associated with perforation and the frequently large size of a mediastinal mature teratoma. We report a rare case of an asymptomatic perforating mediastinal mature teratoma successfully treated with complete thoracoscopic surgery.

**Presentation of case:**

The patient was a 15-year-old girl with an abnormal shadow at the mediastinum seen on chest radiography at a health examination performed at her junior high school; an asymptomatic mediastinal tumour was suspected. Enhanced computed tomography revealed a 6.5 × 3.5-cm tumour associated with the right upper lobe. Thoracoscopic surgery was performed using 4 access ports. Although there were inflammatory changes, hemi-thymectomy and right upper lobectomy were achieved with complete thoracoscopic surgery. The tumour included the skin, sebaceous glands, hair, and pancreatic tissue and was diagnosed as a mature teratoma perforating the right upper lobe.

**Discussion:**

There might be limited inflammatory changes if the patient has few or no symptoms, as in our case. In such cases, the tumour may be excised even if it is large.

**Conclusion:**

We report a rare case of an asymptomatic perforating mediastinal mature teratoma that was successfully treated with complete thoracoscopic surgery. Although infection, inflammatory adhesion, large tumour size, and the need for radical cure are concerns, complete thoracoscopic surgery might be helpful. In cases of few or no symptoms, thoracoscopic surgery is worth challenging.

## Highlights

• Open surgery is usually performed for a perforating mediastinal mature teratoma.
• Asymptomatic perforation involving a teratoma is very rare.
• Complete thoracoscopic surgery was successfully used in this case.
• Complete thoracoscopic surgery might be meaningful in cases of few or no symptoms.

## 1 Introduction

A mediastinal mature teratoma is often diagnosed based on symptoms associated with perforation. A perforating teratoma can cause severe inflammation; therefore, surgery for this tumour is mainly involves open thoracotomy or sternotomy. Complete thoracoscopic surgery is often difficult because of the inflammation associated with perforation and the frequently large size of a mediastinal mature teratoma. Asymptomatic perforation involving a teratoma is very rare.

Herein, we report a rare case of a mediastinal mature teratoma without any symptoms, which showed perforation of the right lung. The teratoma was successfully resected with complete thoracoscopic surgery.

This paper was written according to the SCARE criteria for case reports [1].

## 2 Presentation of case

**Fig. 1:**
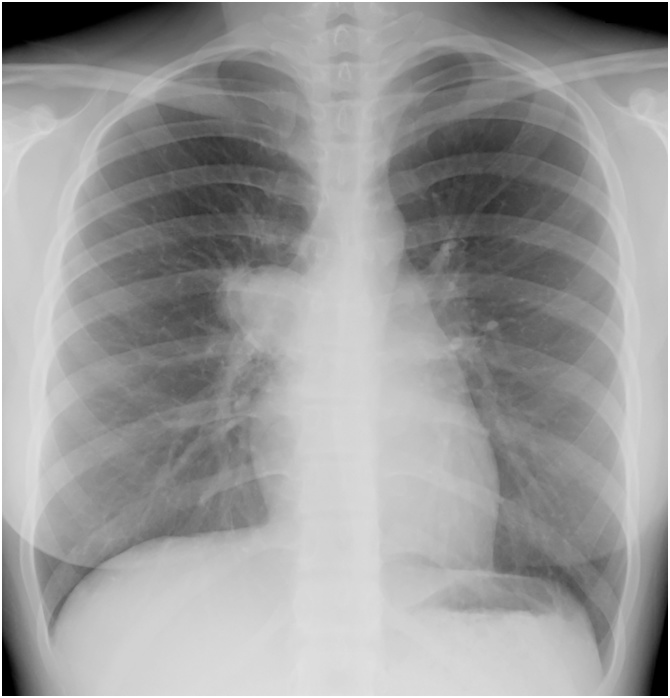
Chest plain radiography shows a 4.0 × 3.5 cm tumour adjacent to the mediastinum.

A 15-year-old female patient was admitted to our hospital because of an abnormal shadow at the mediastinum seen on chest radiography at a health examination performed at her junior high school (Fig. 1). She had two sisters and a brother. Her family including her parents had been healthy and had no inheritable diseases. She had no comorbidity, drug history, or psychosocial history. The first time we met her, she did not have any symptoms, such as cough, fever, or chest pain. Moreover, she had never been aware of those symptoms.

**Fig. 2:**
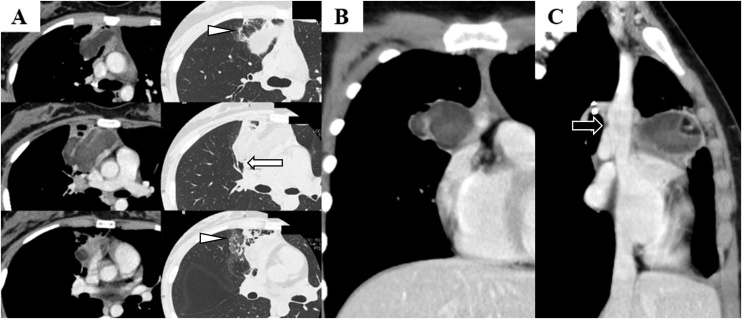
Enhanced computed tomography shows a 6.5 × 3.5-cm tumour with areas of fat density and a thick enhancing nodular wall on the mediastinal aspect. The B^3^ bronchus is obstructed by the tumour (white arrow) and there are ground glass opacities at the right upper lobe around the tumour (white corn). (A. Horizontal view). The tumour is contiguous to the superior vena cava (black arrow), but there are no oppressed vessels. (B. Coronal view and C. sagittal view)

In our hospital, enhanced computed tomography showed 6.5 × 3.5-cm tumour that had areas of fat density and a thick enhancing nodular wall on mediastinal aspect. The B^3^ bronchus was obstructed by the tumour and ground glass opacities were observed at the right upper lobe around the tumour (Fig. 2). Blood examinations showed no inflammatory changes. The white blood cell count was 7140/μL and C-reactive protein level was 0.1 mg/dL. We thought that the tumour was a teratoma, but infectious congenital pulmonary airway malformation or malignancy could not be ruled out. In addition to such uncertainty, the origin of the tumour was unclear the lung or the thymus. We decided to perform thoracoscopic right hemithymo-thymectomy with right upper lobectomy for assuring the surgical margin of both the lung and the thymus.

**Fig. 3:**
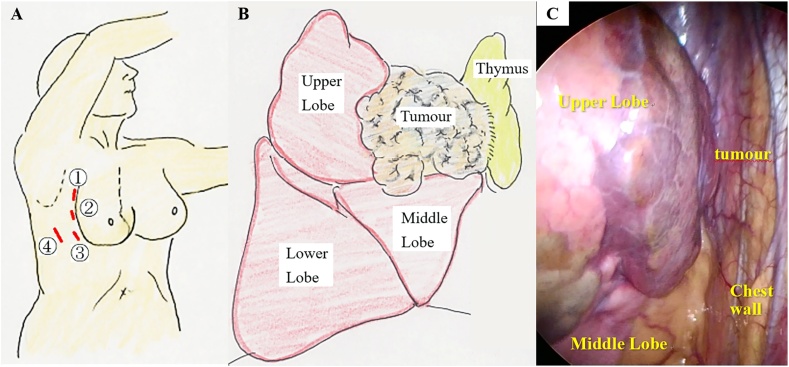
Left hemi-lateral position. Four-port thoracoscopic surgery. Three ports (5 mm) are set along with extension of the inframammary line (respectively 3^rd^, 4^th^, 5^th^ intercostal space). Another port (2 cm) is set on the middle axillary line of the 6^th^ intercostal space. (A. Port setting). Intraoperative thoracic findings. The tumour appears to arise from the right upper lobe and attached to the thymus. (B. Schema) The right upper lobe appears to contain abscess or fat like tissue and does not collapse. (C. Thoracoscopic picture)

In the left hemilateral position, thoracoscopic surgery was performed using 4 access ports (Fig. 3A) by 3 surgeons including 2 specialists at general thoracic surgery. Artificial pneumothorax was used with 8 mmHg CO2 gas supply. The tumour appeared to arise from the right upper lobe and attached to the thymus at the first impression. The tumour had no other adhesion. Almost half of the collapsed right upper lobe had pleural discoloration (Fig. 3B, C). It seemed to be impossible to avoid lobectomy. However, there were limited inflammatory changes in the mediastinum, and the border between the tumour and thymus was unclear. Even then, the tumour origin could not be determined; therefore, we proceeded to perform right hemi-thymothymectomy, following right upper lobectomy as scheduled. The hemi-thymothymectomy was easy; however, because of the inflammatory changes in the peripheral tissues around the pulmonary artery and vein, the membrane around the vessels was peeled with some difficulty. Finally, we completed the hemi-thymothymectomy and right upper lobectomy with complete thoracoscopic surgery in 4 h and 19 min. The total blood loss was 67 mL.

**Fig. 4:**
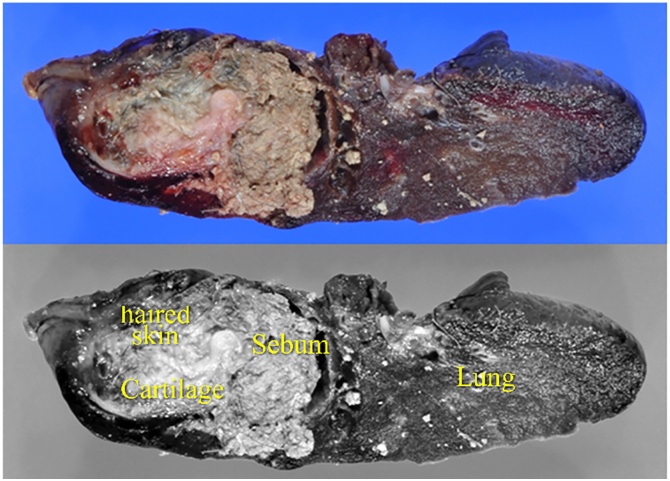
Macroscopically, the tumour is a 5 × 3-cm cystic lesion with sebum, hair, and cartilage.

**Fig. 5:**
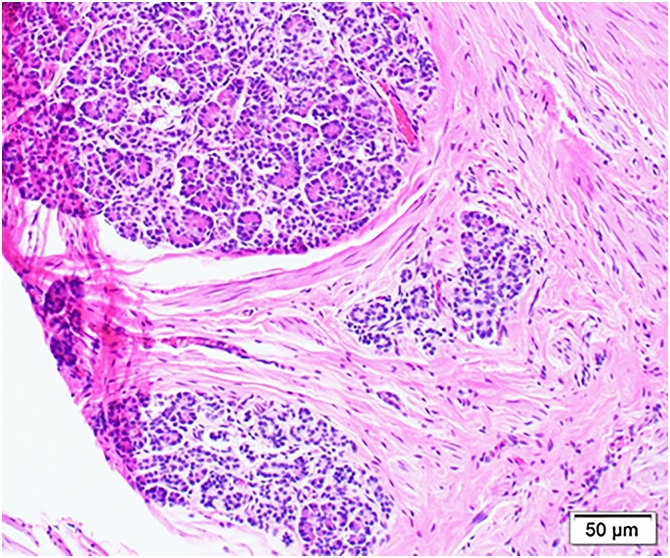
Microscopic findings (haematoxylin and eosin staining). The tumour contains pancreatic tissue.

Macroscopically, the tumour was a 5 × 3-cm cystic lesion containing sebum and hair (Fig. 4). Microscopically, the tumour included skin, sebaceous glands, hair, and pancreatic tissue (Fig. 5) and was covered by a fibrous capsule without any immature or atypical cells. Additionally, the tumour was closely involved with the lung and was adjacent to thymic tissue, without inflammatory changes; therefore, the tumour was diagnosed as a mature teratoma derived from the mediastinum.

The patient was discharged from our hospital on the 5^th^ postoperative day. Two weeks after the surgery, she could go to high school without any problem. There was no recurrence, intrathoracic infection, persistent pain or thoracic deformation 1 year after the surgery.

## 3 Discussion

Mediastinal mature teratomas have been reported to occur in young adults and 50–62% of patients have been shown to present without symptoms [2,3]. On the other hand, 36–41% of patients have been shown to present with symptoms associated with perforation, such as chest pain, fever, haemoptysis and spitting tumour contents [4,5]. Most perforating tumours have been reported to penetrate the lung or bronchus [4,5]. Perforation involving a teratoma usually causes some symptoms and inflammation. Asymptomatic perforation involving a teratoma is very rare. In the English literature, 1 report mentioned the resection of a perforating mature teratoma with complete thoracoscopic surgery [6]. In this case, the tumour had perforated the thoracic cavity, and thoracic effusion provided the opportunity for diagnosis. A mediastinal mature teratoma is often large, and perforation causes inflammation. Therefore, complete thoracoscopic surgery is often difficult. However, there might be limited inflammatory changes if the patient has few or no symptoms, as in our case and the previously reported case. Additionally, in our case, the tumour was soft, and it could be taken out from the thorax. In such a patient with few or no symptoms, the tumour may be taken out even if it is large.

Some mechanisms for perforation have been reported. One mechanism involves inflammation, infection, ischemia, and necrosis caused by the sebaceous component of the tumour and another involves autolysis caused by digestive enzymes derived from the pancreatic or salivary gland component of the tumour [[4], [5], [6], [7], [8], [9]]. In our case, there was a pancreatic gland component, limited inflammation, and no necrosis; therefore, the latter mechanism may have been associated with perforation. However, the reason why inflammatory markers did not elevate in this case was unclear: it could be that the tumour growth was very slow and digestive enzymes excavated the lung little by little.

## 4 Conclusion

In conclusion, we reported a rare case of a perforating mediastinal mature teratoma without any symptoms that was successfully treated with complete thoracoscopic surgery. Although infection, inflammatory adhesion, the large tumour size, and need for a radical cure are concerns, complete thoracoscopic surgery might be helpful. In cases of few or no symptoms, thoracoscopic surgery is worth challenging.

## Conflicts of interest

The authors declare that they have no competing interests.

## Sources of funding

This study was not funded by any sponsor.

## Ethical statement

This study was exempted from ethical approval in University of Yamanashi.

## Consent

Written informed consent was obtained from the patient’s parents for publication of this case report and accompanying images. A copy of the written consent is available for review by the Editor-in-Chief of this journal on request.

## Author contribution

Hiroyasu Matsuoka: Conceptualization, Methodology, Validation, Investigation, Resources, Data Curation, Writing – Original Draft, Visualization

Hirochika Matsubara: Writing – Review & Editing, Supervision

Aya Sugimura: Investigation

Tsuyoshi Uchida: Investigation

Tomofumi Ichihara: Investigation

Hiroyuki Nakajima: Project Administration

## Registration of research studies

NA.

## Guarantor

Hirochika Matsubara is the Guarantor in this case report.

Authorship declaration

All authors are in agreement with the content of the manuscript.

## Provenance and peer review

Not commissioned, externally peer reviewed.
